# Constructal Optimizations of Line-to-Line Vascular Channels with Turbulent Convection Heat Transfer

**DOI:** 10.3390/e24070999

**Published:** 2022-07-19

**Authors:** Daoguang Lin, Zhihui Xie, Gang Nan, Pan Jiang, Yanlin Ge

**Affiliations:** 1 College of Power Engineering, Naval University of Engineering, Wuhan 430033, China; ldg774119975@163.com (D.L.); gang_nan@163.com (G.N.); jiangpan202109@163.com (P.J.); 2 School of Energy and Electromechanic Engineering, Hunan University of Humanities, Science and Technology, Loudi 417000, China; 3 Institute of Thermal Science and Power Engineering, Wuhan Institute of Technology, Wuhan 430205, China; geyali9@hotmail.com; 4 School of Mechanical & Electrical Engineering, Wuhan Institute of Technology, Wuhan 430205, China

**Keywords:** constructal theory, entropy generation minimization principle, turbulence, line-to-line vasculature, generalized thermodynamic optimization

## Abstract

The multi-scale line-to-line vascular channels (LVCs) widely exist in nature because of their excellent transmission characteristics. In this paper, models of LVCs with turbulent convection heat transfer are established. Based on constructal theory and the entropy generation minimization principle, the constructal optimizations of LVCs with any order are conducted by taking the angles at bifurcations as the optimization variables. The heat flux on the channel wall per unit length is fixed and uniform. The areas occupied by vasculature and the total volumes of channels are fixed. The analytical expressions of the optimal angles, dimensionless total entropy generation rate and entropy generation number (EGN) of LVCs with any order versus dimensionless mass flow rate are obtained, respectively. The results indicate that the dimensionless total entropy generation rate of LVCs with any order can be significantly decreased by optimizing the angles of LVCs, which is significantly more when the order of LVCs is higher. As the dimensionless mass flow rate increases, the optimal angles of LVCs with any order remain unchanged first, then the optimal angles at the entrance (root) increase, and the other optimal angles decrease continuously and finally tend to the respective stable values. The optimal angles of LVCs continue to increase from the entrance to the outlet (crown), i.e., the LVCs with a certain order gradually spread out from the root to the crown. The dimensionless total entropy generation rate and EGN of LVCs first decrease and then increase with the growth of the dimensionless mass flow rate. There is optimal dimensionless mass flow rate, making the dimensionless total entropy generation rate and the EGN reach their respective minimums. The results obtained herein can provide some new theoretical guidelines of thermal design and management for the practical applications of LVCs.

## 1. Introduction

Electronic devices are widely used in various engineering fields. The heat generation rate of electronic device per unit volume increases sharply with the smaller volume and larger power and higher integration, which poses new challenges to the thermal control of electronic devices, and the thermal design optimization for electronics cooling is one of the advisable and effective ways to meet these challenges [1,2,3,4]. The branching structures, such as tree branches, leaf veins, bronchial trees, blood vessels and rivers, exist widely in nature, which provide some meaningful references for liquid-cooled microchannel designs for the thermal management of electronic devices.

Constructal theory was proposed by Bejan in 1996 [5,6], and the basic gist of constructal theory is constructal law, which can be described as “For a finite-size flow system to persist in time (to live), its configuration must change in time such that it provides easier and easier access to its currents”, which explained the profound reasons for the formation of various flow structures in nature and social fields. The constructal theory can be used to seek the optimal designs of devices and systems, and thus it has also been named as a new philosophy of geometry. Since it was proposed, constructal theory has been widely used in various fields [7,8,9,10,11,12,13,14,15,16,17,18,19,20,21,22]. As one of the main fields, it has opened up new research on the optimizations of heat and mass transfer, such as heat source [23,24,25,26], heat conduction [27,28,29,30], convection heat transfer [31,32,33] and heat exchange equipment [34,35,36,37,38]. 

The vascular channels are composed of many multi-scale branching channel units and have excellent transmission characteristics (which are expected to effectively resolve the technical bottleneck problem of heat dissipation for electronic devices), and they have become one of the hottest objects in constructal design [39,40,41,42,43,44,45]. Lorente and Bejan [39] proposed the novel dendritic flow architecture called line-to-line trees in 2006, and they optimized the performance of line-to-line trees by minimizing the maximum pressure difference and found that it was far superior to the traditional parallel vasculature. 

Kim et al. [46] optimized the design of tree-shape vasculature by minimizing the maximum pressure difference, and the optimal shape and optimal diameter ratio of a vascular network were obtained. Wang et al. [47,48] indicated that the flow performance of multi-scale vascular channels was better than that of single-scale vascular channels. Lee et al. [49] optimized the vascular channels in the square areas by minimizing the pressure drop. The results showed that the non-uniform structure provided greater overall flow access. 

Liu et al. [50] optimized the design of a T-Y-shaped vascular channel by minimizing the pressure difference. Miguel [51] optimized the bifurcated vascular channels by taking the minimum flow resistance as the optimization objective. The relationships between the size of the parent and daughter tubes at bifurcations, and the branching angles of the bifurcations were obtained. Hu et al. [52] studied H-shaped vascular channels and found that the vascular network with multiple primary channels had better transmission performance. 

Jing and Song [53] compared the thermal and hydraulic performances of two tree-like networks with the fixed surface areas and fixed volumes. The results showed that the optimal channel diameter ratio to reach a minimum hydraulic resistance varied with surface area constraint and volume constraint. Lu et al. [54] studied the relationships between the branching level and the cooling performance of Y-shaped liquid cooling heat sink and obtained the effects of branching level on the pressure loss and maximum temperature. The above studies of optimization for vascular channels were mainly conducted with the single objective of flow or heat transfer performance.

Reducing thermal resistance often increases the flow resistance. It is necessary to consider the two contradictory objectives, i.e., heat transfer performance and hydraulic performance, to make the optimization results more in line with the practical needs. The objective of entropy generation minimization is to seek the minimization of thermodynamic irreversibility, which can reflect the comprehensive performance of the heat transfer and hydraulics of a system and is widely used in various heat transfer process and system optimizations. 

Wechsatol et al. [55] studied the effect of junction losses on the optimized geometry of tree-shaped flows. Zimparov et al. [56,57] optimized the H-shaped vascular channels in the rectangular domain and the Y-shaped vascular channels in the disk domain under laminar flow by taking the total entropy generation rate (EGR) of heat transfer and fluid flow as the objective. Furthermore, under turbulent flow, the designs of T-shaped and H-shaped vasculatures were optimized [58,59]. 

Feng et al. [60] optimized the asymmetric vasculature in a disc-shaped body by minimizing dimensionless total entropy generation rate and dimensionless entropy generation ratio, respectively. Xie et al. [41] optimized the LVCs with fixed vascular areas and total channel volumes by taking the minimum dimensionless total entropy generation rate per unit load of heat transfer as the objective. Feng et al. [61] optimized the design of X-shaped vasculature and indicated that the EGR of X-shaped vascular channels was smaller than that of H-shaped vascular channels under the same geometric constraints. 

Miguel [62,63] optimized the Y-shaped vasculature, the optimum ratios of channel diameters and channel lengths, and the optimum angle between branches were investigated. Shi et al. [64] optimized the LVCs with convection heat transfer under laminar conditions by taking the angles as the optimization variables and the minimum EGR as the optimization objective. The results showed that the EGR of the LVCs decreases when the angle freedom increases with the constant dimensionless mass flow rate. When the order increases, the EGR of the LVCs increases but the EGN decreases. Both the EGR and the EGN increase with the increase of the dimensionless mass flow rate.

The branching angles at bifurcations of LVCs have great influences on both fluid flow and heat transfer, which need to be further studied; however, there is no study on the angle optimization for multi-scale LVCs with turbulent convection heat transfer by combining method of constructal theory and entropy generation minimization principle in the open literatures. In this paper, the analytical method is used to optimize the geometric structures of multi-scale LVCs with turbulent convection heat transfer based on entropy generation minimization principle. 

The expressions of the dimensionless total entropy generation rate and EGN of LVCs with any order versus dimensionless mass flow rate are deduced by taking the linear heat flux as a constant and the branching angles at bifurcations as the optimization variables with the constraints of fixed areas and fixed total channel volumes. Constructal optimizations of LVCs with turbulent convection heat transfer are conducted by minimizing the dimensionless total entropy generation rate and EGN. It is expected to provide some new theoretical supports for the practical applications of a line-to-line vascular system.

## 2. Geometric Model of LVCs

**Figure 1:**
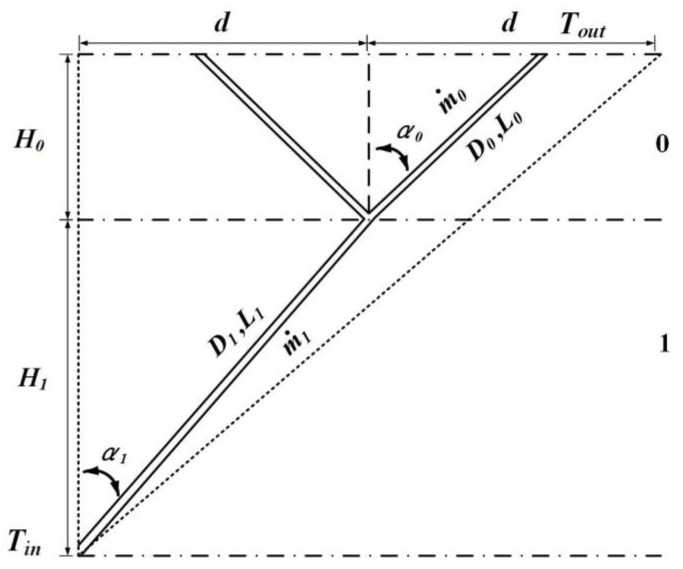
The first order LVCs with turbulent convection heat transfer.

The LVCs are a self-similar branching network composed of circular section channels. The *i*th channel with two branches with the same diameter and length assemble into (*i* + 1)th-level channel. The first order LVCs with turbulent convection heat transfer are shown in Figure 1. The fluid flows into the root of LVCs and flows out from the crown of LVCs, and convection heat transfer occurs between the inner walls of LVCs and the fluid. For each level, the length is *L_i_*, and the vertical distance is *H_i_*. The temperature of fluid is *T_in_* (K) at the entrance and *T_out_* (K) at the outlet. 

The thermal conductivity of fluid is *k* (W·(m·K)^−1^), the density is ρ (kg·m^−3^), the kinematic viscosity is ν (m·s^−2^), and the mass flow rate is $\dot{m}$ (kg·s^−1^) (${\dot{m}}_{i}{}_{+1}=2{\dot{m}}_{i}$). The heat flow on the each axially uniform channel of circular cross-section surface is fixed per unit length, i.e., the linear heat flux $q^{\prime }$ (W·m^−1^) of channel is taken as a constant. The flow is assumed to be fully developed turbulence. The local pressure losses at the junctions of LVCs are negligible.

The area occupied by vasculature and the total channel volumes can be expressed as follows [39,64]:

(1) $$A_{n}=2^{n-1}d\sum_{i=0}^{n}H_{i}$$

(2) $$V_{n}=\frac{\pi }{4}\sum_{i=0}^{n}2^{n-i}D_{i}{}^{2}L_{i}$$

The length and vertical distance of the *i*th order vascular channels are:

(3) $$L_{i}=\frac{2^{i-1}d}{\sin\alpha_{i}}$$

(4) $$H_{i}=L_{i}\cos\alpha_{i}$$

Under turbulent flow conditions, assuming that the diameters of channels at any two adjacent vascular channels follow $D_{i+1}=2^{2/5}D_{i}$ [55,58]. Thus, one can obtain from Equation (2):

(5) $$D_{0}={\left(\frac{\pi \sum_{i=0}^{n}2^{\frac{5n-i-10}{5}}L_{i}}{V}\right)}^{-1/2}$$

Combining Equations (1), (3) and (4), one can obtain:

(6) $${\left(d/A^{1/2}\right)}_{n}={\left(\sum_{i=0}^{n}2^{n}{}^{+i-2}\cot\alpha_{i}\right)}^{-1/2}$$

When the linear heat flux is constant and the cooling fluid flow is fully developed turbulence, the EGR in *i*th channel with convection heat transfer is [58]:

(7) $${\dot{S}}_{gen,i}=\frac{q_{i}\Delta T}{T_{i}^{2}}\frac{1}{\left(1+\frac{\Delta T_{x}^{i}}{T_{i}}\right)}+\frac{32{\dot{m}}_{i}{}^{3}f_{i}L_{i}}{\rho^{2}\pi^{2}T_{i}D_{i}^{5}}\frac{\ln(1+\frac{\Delta T_{x}^{i}}{T_{i}})}{\frac{\Delta T_{x}^{i}}{T_{i}}}$$

assuming that:

(8) fi=0.046Rei−1/5=0.023π1/523/5μ1/5(Di/m˙i)1/5Nu=0.023Rei4/5Pr2/5=0.023Pr2/5(m˙i/Di)4/5/(πμ)4/5

Thus, the EGR in *i*th channel with convection heat transfer is:

(9) $${\dot{S}}_{gen,i}=\frac{{q^{\prime }}^{2}\mu^{4/5}L_{i}}{0.023\pi^{1/5}2^{8/5}kT^{2}{\mathrm{Pr}}^{2/5}}{\left(\frac{D_{i}}{{\dot{m}}_{i}}\right)}^{4/5}+\frac{0.023\mu^{1/5}2^{28/5}L_{i}}{\rho^{2}\pi^{9/5}T}{\left(\frac{{\dot{m}}_{i}^{7}}{D_{i}^{12}}\right)}^{2/5}$$

Equation (9) can be presented in dimensionless form:

(10) $${\tilde{S}}_{gen,i}=\frac{T{\dot{S}}_{gen,i}}{q^{\prime }\tilde{q^{\prime }}A^{1/2}}=\frac{D_{i}{}^{4/5}}{A^{2/5}M^{4/5}}{\tilde{L}}_{i}+B_{0}M^{14/5}\frac{\mu^{3}A^{7/5}}{\rho^{2}kTD_{i}^{24/5}}{\tilde{L}}_{i}$$

where $\tilde{q^{\prime }}=q^{\prime }/(0.023\pi^{1/5}2^{8/5}kT)$ is the dimensionless linear heat flux, $M=\dot{m}{\mathrm{Pr}}^{1/2}/(\mu A^{1/2})$ is the dimensionless mass flow rate, $B_{0}=2^{4}/({\tilde{q^{\prime }}}^{2}\pi^{2}Pr^{7/5})$ is the coefficient, and ${\tilde{L}}_{i}=L_{i}/A^{1/2}$ is the dimensionless channel length.

## 3. Constructal Optimizations of LVCs with Minimum EGR

### 3.1. Constructal Optimizations of the First Order LVCs

The first order LVCs with the angles as optimization variables are shown in Figure 1. The dimensionless total entropy generation rate can be obtained by combining Equation (10) with the geometric structure characteristics:

(11) $${\tilde{S}}_{gen,1}=\frac{1}{M^{4/5}A^{2/5}}(2^{9/5}D_{0}{}^{4/5}{\tilde{L}}_{0}+D_{1}{}^{4/5}{\tilde{L}}_{1})+B_{0}M^{14/5}\frac{\mu^{3}A^{7/5}}{kT\rho^{2}}(2^{-9/5}\frac{{\tilde{L}}_{0}}{D_{0}^{24/5}}+\frac{{\tilde{L}}_{1}}{D_{1}^{24/5}})$$

According to Equations (5) and (11), one can obtain:

(12) $$\begin{array}{l} {\tilde{S}}_{gen,1}=\frac{V^{2/5}}{\pi^{2/5}A^{3/5}}\frac{1}{M^{4/5}}(2^{-1}{\tilde{L}}_{0}+2^{-6/5}{\tilde{L}}_{1}{)}^{-2/5}(2^{9/5}{\tilde{L}}_{0}+2^{8/25}{\tilde{L}}_{1})\\ +B_{0}M^{14/5}\frac{\mu^{3}\pi^{12/5}A^{13/5}}{\rho kTV^{12/5}}(2^{-1}{\tilde{L}}_{0}+2^{-6/5}{\tilde{L}}_{1}{)}^{12/5}(2^{-9/5}{\tilde{L}}_{0}+2^{-48/25}{\tilde{L}}_{1}) \end{array}$$

By further simplifying Equation (12), one can obtain:

(13) $$\begin{array}{l} {\tilde{S}}^{*}{}_{gen,1}=\frac{\pi^{2/5}A^{3/5}}{V^{2/5}}{\tilde{S}}_{gen,1}=\frac{1}{M^{4/5}}(2^{-1}{\tilde{L}}_{0}+2^{-6/5}{\tilde{L}}_{1}{)}^{-2/5}(2^{9/5}{\tilde{L}}_{0}+2^{8/25}{\tilde{L}}_{1})\\ +B_{0}^{\prime }M^{14/5}(2^{-1}{\tilde{L}}_{0}+2^{-6/5}{\tilde{L}}_{1}{)}^{12/5}(2^{-9/5}{\tilde{L}}_{0}+2^{-48/25}{\tilde{L}}_{1}) \end{array}$$

where $B_{0}^{\prime }=\frac{2^{4}}{{\tilde{q^{\prime }}}^{2}{\mathrm{Pr}}^{7/5}}\frac{\mu^{3}\pi^{4/5}A^{16/5}}{\rho kTV^{14/5}}$.

Substituting Equations (3) and (6) into Equation (13):

(14) $$\begin{array}{l} {\tilde{S}}^{*}{}_{gen,1}=\frac{1}{M^{4/5}}{\left(2^{-2}\csc\alpha_{0}+2^{-6/5}\csc\alpha_{1}\right)}^{-2/5}(2^{4/5}\csc\alpha_{0}+2^{8/25}\csc\alpha_{1})(2^{-1}\cot\alpha_{0}\\ +\cot\alpha_{1}{)}^{-3/10}+B_{0}^{\prime }M^{14/5}{\left(2^{-2}\csc\alpha_{0}+2^{-6/5}\csc\alpha_{1}\right)}^{12/5}(2^{-14/5}\csc\alpha_{0}\\ +2^{-48/25}\csc\alpha_{1}){\left(2^{-1}\cot\alpha_{0}+\cot\alpha_{1}\right)}^{-17/10} \end{array}$$

According to Equation (14), when the dimensionless mass flow rate M and the dimensionless coefficient $B_{0}^{\prime }$ are constant, the dimensionless total entropy generation rate is only related to the two angles $\alpha_{0}$ and $\alpha_{1}$. When $B_{0}^{\prime }$ = 1 (for the convenience of calculation, $B_{0}^{\prime }$ is uniformly taken as 1 for the following calculations) and M=1, the optimal angles $\alpha_{\mathrm{opt},0}=72.6{}^{\circ}$ and $\alpha_{\mathrm{opt},1}=27.2{}^{\circ}$ can be obtained. The dimensionless total entropy generation rate decreases by 10.65% compared with that of the first order LVCs with fixed angles ($\alpha_{i}=45.0{}^{\circ}$).

However, there are two extreme cases in the optimization process:

When $\frac{1}{M^{4/5}}\ll B_{0}^{\prime }M^{14/5}$, i.e., $B_{0}^{\prime }M^{18/5}\gg 1$, the dimensionless total entropy generation rate can be expressed as:

(15) $$\begin{array}{l} {\tilde{S}}^{*}{}_{gen,1}=B_{0}^{\prime }B_{0}^{\prime }M^{14/5}{\left(2^{-2}\csc\alpha_{0}+2^{-6/5}\csc\alpha_{1}\right)}^{12/5}(2^{-14/5}\csc\alpha_{0}\\ +2^{-48/25}\csc\alpha_{1}){\left(2^{-1}\cot\alpha_{0}+\cot\alpha_{1}\right)}^{-17/10} \end{array}$$

The optimal angles $\alpha_{\mathrm{opt},0}=49.7{}^{\circ}$ and $\alpha_{\mathrm{opt},1}=42.5{}^{\circ}$ can be obtained by calculation.

When $\frac{1}{M^{4/5}}\gg B_{0}^{\prime }M^{14/5}$, i.e., $B_{0}^{\prime }M^{18/5}\ll 1$, the dimensionless total entropy generation rate can be expressed as:

(16) $$\begin{array}{l} {\tilde{S}}^{*}{}_{gen,1}=\frac{1}{M^{4/5}}{\left(2^{-2}\csc\alpha_{0}+2^{-6/5}\csc\alpha_{1}\right)}^{-2/5}(2^{4/5}\csc\alpha_{0}\\ +2^{8/25}\csc\alpha_{1}){\left(2^{-1}\cot\alpha_{0}+\cot\alpha_{1}\right)}^{-3/10} \end{array}$$

The optimal angles $\alpha_{\mathrm{opt},0}=77.8{}^{\circ}$ and $\alpha_{\mathrm{opt},1}=20.7{}^{\circ}$ can be obtained by calculation.

### 3.2. Constructal Optimizations of the Second Order LVCs

**Figure 2:**
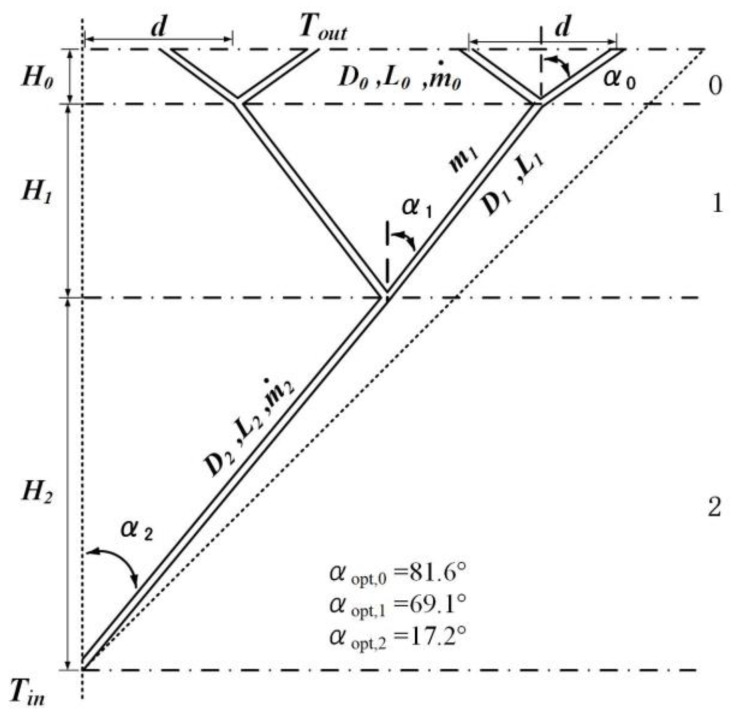
The second order LVCs with turbulent convection heat transfer.

The second order LVCs with the angles as optimization variables are shown in Figure 2. Combining Equations (3), (5), (6) and (10) with the geometric structure characteristics of the second order LVCs, the dimensionless total entropy generation rate can be written as:

(17) $$\begin{array}{l} {\tilde{S}}^{*}{}_{gen,2}=\frac{1}{M^{4/5}}(2^{13/5}\csc\alpha_{0}+2^{53/25}\csc\alpha_{1}+2^{41/25}\csc\alpha_{2})(2^{-1}\csc\alpha_{0}\\ +2^{-1/5}\csc\alpha_{1}+2^{3/5}\csc\alpha_{2}{)}^{-2/5}{\left(\cot\alpha_{0}+2\cot\alpha_{1}+2^{2}\cot\alpha_{2}\right)}^{-3/10}\\ +B_{0}^{\prime }M^{14/5}(2^{-23/5}\csc\alpha_{0}+2^{-93/25}\csc\alpha_{1}+2^{-71/25}\csc\alpha_{2})(2^{-1}\csc\alpha_{0}\\ +2^{-1/5}\csc\alpha_{1}+2^{3/5}\csc\alpha_{2}{)}^{12/5}{\left(\cot\alpha_{0}+2\cot\alpha_{1}+2^{2}\cot\alpha_{2}\right)}^{-17/10} \end{array}$$

According to Equation (17), when the dimensionless mass flow rate M is constant, the dimensionless total entropy generation rate is only related to the three angles $\alpha_{0}$, $\alpha_{1}$ and $\alpha_{2}$. The results show that, when the dimensionless mass flow rate M=1, the optimal angles $\alpha_{\mathrm{opt},0}=81.6{}^{\circ}$, $\alpha_{\mathrm{opt},1}=69.1{}^{\circ}$ and $\alpha_{\mathrm{opt},2}=17.2{}^{\circ}$. The dimensionless total entropy generation rate decreases by 24.54% compared with that of the second order LVCs with fixed angles ($\alpha_{i}=45.0{}^{\circ}$).

### 3.3. Constructal Optimizations of the Third and Higher Order LVCs

**Figure 3:**
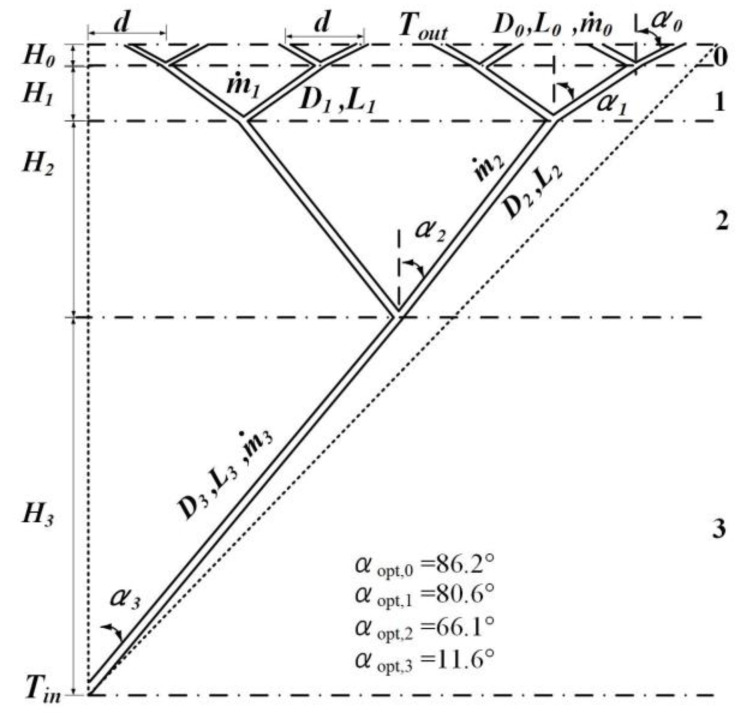
The third order LVCs with turbulent convection heat transfer. The numbers 1–3 in the figure express the (1–3)th-level channels.

The third order LVCs with the angles as optimization variables are shown in Figure 3. Combining Equations (3), (5), (6) and (10) with the geometric characteristics of the third order LVCs, the dimensionless total entropy generation rate of the third order LVCs with turbulent convection heat transfer can be obtained:

(18) $$\begin{array}{l} {\tilde{S}}^{*}{}_{gen,3}=\frac{1}{M^{4/5}}(2^{22/5}\csc\alpha_{0}+2^{98/25}\csc\alpha_{1}+2^{86/25}\csc\alpha_{2}+2^{74/25}\csc\alpha_{3})\\ \times {\left(\csc\alpha_{0}+2^{4/5}\csc\alpha_{1}+2^{8/5}\csc\alpha_{2}+2^{12/5}\csc\alpha_{3}\right)}^{-2/5}{\left(2\cot\alpha_{0}+2^{2}\cot\alpha_{1}+2^{3}\cot\alpha_{2}+2^{4}\cot\alpha_{3}\right)}^{-3/10}\\ +B_{0}^{\prime }M^{14/5}(2^{-32/5}\csc\alpha_{0}+2^{-138/25}\csc\alpha_{1}+2^{-116/25}\csc\alpha_{2}+2^{-94/25}\csc\alpha_{3})\\ {\left(\csc\alpha_{0}+2^{4/5}\csc\alpha_{1}+2^{8/5}\csc\alpha_{2}+2^{12/5}\csc\alpha_{3}\right)}^{12/5}{\left(2\cot\alpha_{0}+2^{2}\cot\alpha_{1}+2^{3}\cot\alpha_{2}+2^{4}\cot\alpha_{3}\right)}^{-17/10} \end{array}$$

According to Equation (18), when the dimensionless mass flow rate *M* is constant, the dimensionless total entropy generation rate is only related to the four angles $\alpha_{0}$, $\alpha_{1}$, $\alpha_{2}$ and $\alpha_{3}$. When the dimensionless mass flow rate M=1, the optimal angles $\alpha_{\mathrm{opt},0}=86.2{}^{\circ}$, $\alpha_{\mathrm{opt},1}=80.6{}^{\circ}$, $\alpha_{\mathrm{opt},2}=66.1{}^{\circ}$ and $\alpha_{\mathrm{opt},3}=11.6{}^{\circ}$. The dimensionless total entropy generation rate decreases by 43.75% compared with that of the third order LVCs with fixed angles ($\alpha_{i}=45.0{}^{\circ}$).

Combining Equations (3), (5), (6) and (10) with geometrical characteristics of the LVCs, the dimensionless total entropy generation rate of *n*th order LVCs with turbulent convection heat transfer can be further derived as:

(19) $$\begin{array}{l} {\tilde{S}}^{*}{}_{gen,n}=\frac{1}{M^{4/5}}(\sum_{i=0}^{n}2^{\frac{45n-12i-25}{25}}\csc\alpha_{i}){\left(\sum_{i=0}^{n}2^{\frac{5n+4i-15}{5}}\csc\alpha_{i}\right)}^{-2/5}{\left(\sum_{i=0}^{n}2^{n-2+i}\cot\alpha_{i}\right)}^{-3/10}\\ +B_{0}^{\prime }M^{14/5}(\sum_{i=0}^{n}2^{\frac{22i-45n-25}{25}}\csc\alpha_{i}){\left(\sum_{i=0}^{n}2^{\frac{5n+4i-15}{5}}\csc\alpha_{i}\right)}^{12/5}{\left(\sum_{i=0}^{n}2^{n-2+i}\cot\alpha_{i}\right)}^{-17/10} \end{array}$$

**Figure 4:**
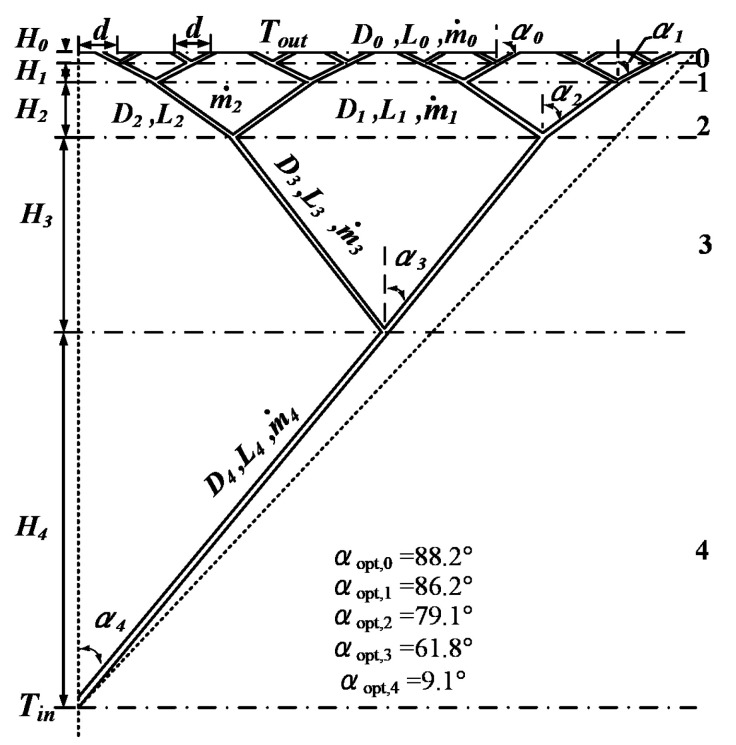
The fourth order LVCs with turbulent convection heat transfer. The numbers 1–4 in the figure express the (1–4)th-level channels.

The fourth order LVCs with the angles as optimization variables are shown in Figure 4. When M=1, the optimal angles $\alpha_{\mathrm{opt},0}=88.2{}^{\circ}$, $\alpha_{\mathrm{opt},1}=86.2{}^{\circ}$, $\alpha_{\mathrm{opt},2}=79.1{}^{\circ}$, $\alpha_{\mathrm{opt},3}=61.8{}^{\circ}$ and $\alpha_{\mathrm{opt},4}=9.1{}^{\circ}$ can be obtained from Equation (19).

When M=1, the optimal angles, $\alpha_{\mathrm{opt},0}=88.8{}^{\circ}$, $\alpha_{\mathrm{opt},1}=88.8{}^{\circ}$, $\alpha_{\mathrm{opt},2}=85.2{}^{\circ}$, $\alpha_{\mathrm{opt},3}=77.1{}^{\circ}$, $\alpha_{\mathrm{opt},4}=61.6{}^{\circ}$ and $\alpha_{\mathrm{opt},5}=6.6{}^{\circ}$ of the fifth order LVCs, can be obtained by further calculation from Equation (19). The dimensionless total entropy generation rate of the fourth and fifth order LVCs decreases by 66.99% and 93.67% compared with that with fixed angles ($\alpha_{i}=45.0{}^{\circ}$), respectively.

By optimizing the angle freedom of vascular channels, the dimensionless total entropy generation rate of LVCs with any order can be significantly decreased. When the order of LVCs is higher, the dimensionless total entropy generation rate is decreased significantly more.

## 4. Effects of the Dimensionless Mass Flow Rate on Constructal Optimizations, Dimensionless Total Entropy Generation Rate and EGN

### 4.1. Effects of the Dimensionless Mass Flow Rate on Constructal Optimizations

**Figure 5:**
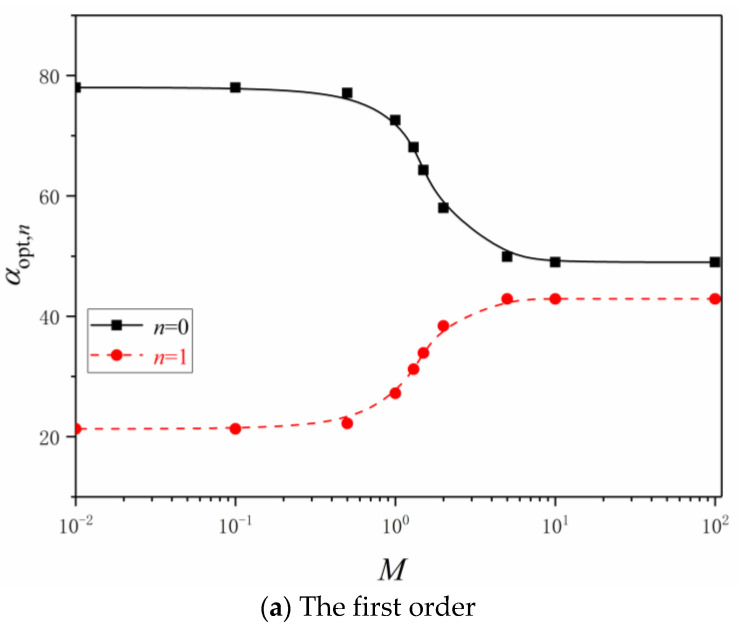

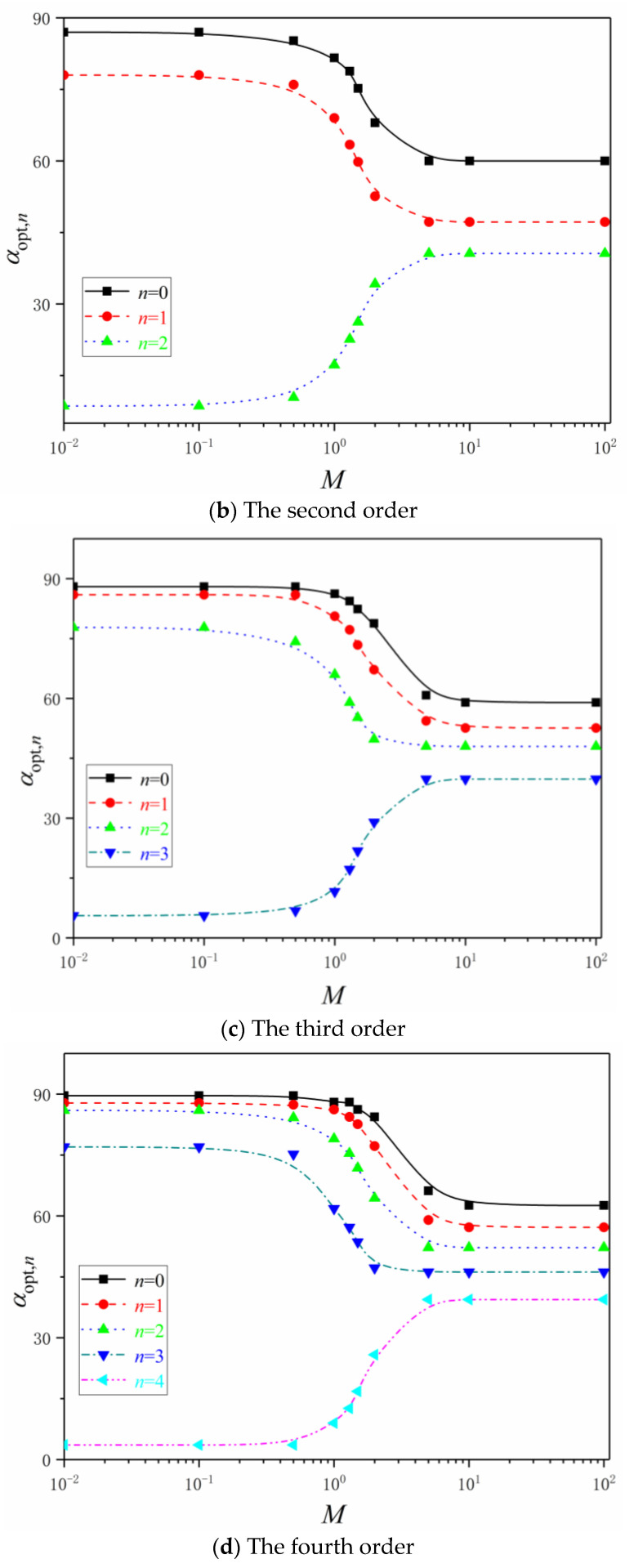

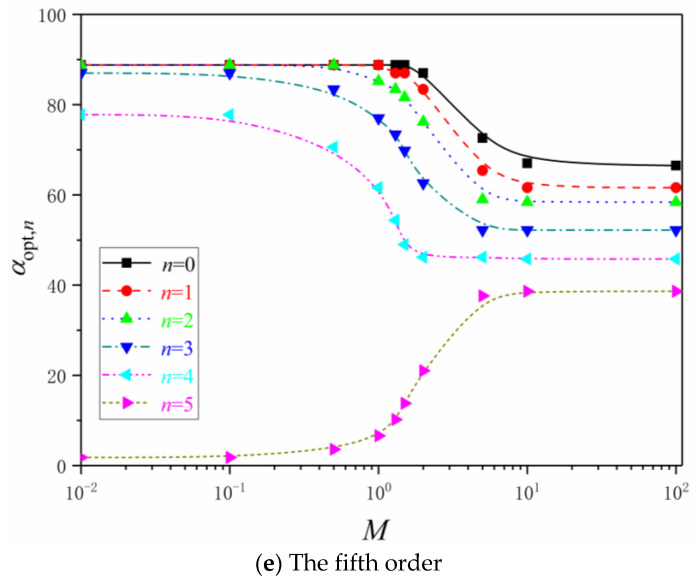
Effects of the dimensionless mass flow rate on the optimal angles.

The influences of the dimensionless mass flow rate on the constructal optimizations of the first to fifth order LVCs are shown in Figure 5. When the dimensionless mass flow rate increases, the optimal angles of LVCs with any order first remain unchanged, and then the optimal angle $\alpha_{\mathrm{opt},i}$ at the entrance increases, the other optimal angles decrease continuously and finally tend to respective stable values. 

The optimal angles of LVCs continue to increase from the entrance to the outlet, that is, the LVCs with a certain order gradually spread out from the root to the crown. When the dimensionless mass flow rate is large or small enough, the optimal angles of LVCs with any order remain unchanged versus the change of the dimensionless mass flow rate. The characteristics of $\alpha_{\mathrm{opt},i}$ versus M are listed in Table 1. When the dimensionless mass flow is constant, the optimal angles of LVCs at the entrance decrease and the optimal angles of LVCs at the outlet increase as the order of LVC increases.

### 4.2. Effects of the Dimensionless Mass Flow Rate on the Dimensionless Total Entropy Generation Rate

**Figure 6:**
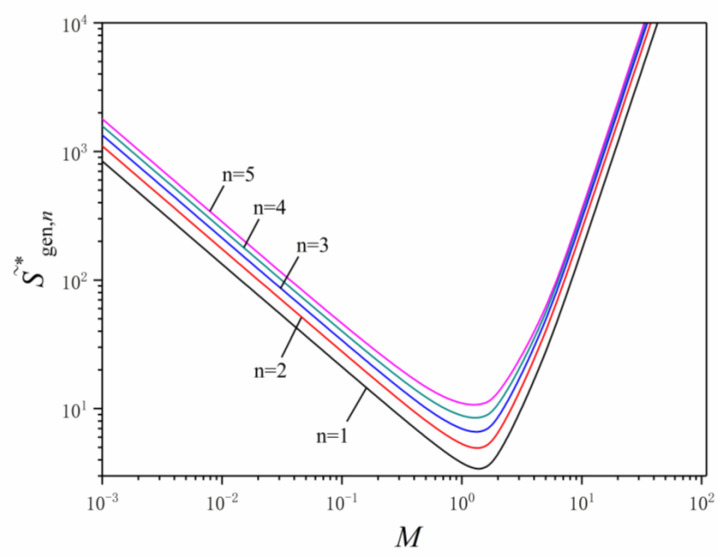
Effects of the dimensionless mass flow rate on the dimensionless total entropy generation rate.

When the dimensionless coefficient $B_{0}^{\prime }$=1, the influences of the dimensionless mass flow rate on the dimensionless total entropy generation rate are shown in Figure 6. For any *n*, the dimensionless total entropy generation rate first decreases and then increases sharply with the growth of the dimensionless mass flow rate M, this change is attributed to the sharp increase of EGR produced by fluid heat exchange when the mass flow is small and the sharp increase of EGR produced by fluid flow when the mass flow is large. When the dimensionless mass flow rate M is between 1 and 2, there are optimal dimensionless mass flow rates that can make the dimensionless total entropy generation rate with any order to reach their respective minimums. 

The dimensionless total entropy generation rate of the first order LVCs is the smallest, and the fifth order LVCs is the largest, the dimensionless total entropy generation rate of LVCs increases gradually as the order of LVC increases. When the dimensionless mass flow rate M is greater than 2, the differences of the dimensionless total entropy generation rate among the first to fifth order LVCs are slightly smaller than dimensionless mass flow rate M less than 2. 

The characteristics of ${\tilde{S}}^{*}{}_{gen,n}$ versus M and $\alpha_{i}$ are listed in Table 2. The dimensionless total entropy generation rate of the first to fifth order LVCs with optimal angles $\alpha_{opt,i}$ significantly decrease compared with that with fixed angles $\alpha_{i}$, which is significantly more when the order of LVCs is higher. When the angles $\alpha_{i}$ of LVCs are fixed, the dimensionless total entropy generation rate of LVCs increases as the order of LVCs increase. 

### 4.3. Effects of the Dimensionless Mass Flow Rate on EGN

Furthermore, EGN (EGR of per unit heat transfer rate) is introduced to study the turbulent convection heat transfer performance of LVCs. The total dimensionless heat transfer rate of *n*th order LVCs can be expressed as:

(20) $${\tilde{q}}_{n}=q/({q^{\prime }}_{}A^{1/2})=q^{\prime }\sum_{i=0}^{n}2^{n-i}L_{i}/(q^{\prime }A^{1/2})$$

Combining Equations (19) and (20) with the geometric structure characteristics of LVCs, the EGN of *n*th order LVCs can be obtained as follows:


(21) $$\begin{array}{l} N_{s,n}=\frac{{\tilde{S}}^{*}{}_{gen,n}}{{\tilde{q}}_{n}}=\frac{1}{M^{4/5}}(\sum_{i=0}^{n}2^{\frac{45n-12i-25}{25}}\csc\alpha_{i}){\left(\sum_{i=0}^{n}2^{\frac{5n+4i-15}{5}}\csc\alpha_{i}\right)}^{-2/5}{\left(\sum_{i=0}^{n}2^{n-2+i}\cot\alpha_{i}\right)}^{-3/10}\\ {\left[\sum_{i=0}^{n}2^{n-i}(2^{i-1}\csc\alpha_{i}{\left(\sum_{i=0}^{n}2^{i+n-2}\cot\alpha_{i}\right)}^{-1/2})\right]}^{-1}+B_{0}^{\prime }M^{14/5}(\sum_{i=0}^{n}2^{\frac{22i-45n-25}{25}}\csc\alpha_{i}){\left(\sum_{i=0}^{n}2^{\frac{5n+4i-15}{5}}\csc\alpha_{i}\right)}^{12/5}\\ {\left(\sum_{i=0}^{n}2^{n-2+i}\cot\alpha_{i}\right)}^{-17/10}{\left[\sum_{i=0}^{n}2^{n-i}(2^{i-1}\csc\alpha_{i}{\left(\sum_{i=0}^{n}2^{i+n-2}\cot\alpha_{i}\right)}^{-1/2})\right]}^{-1} \end{array}$$

**Figure 7:**
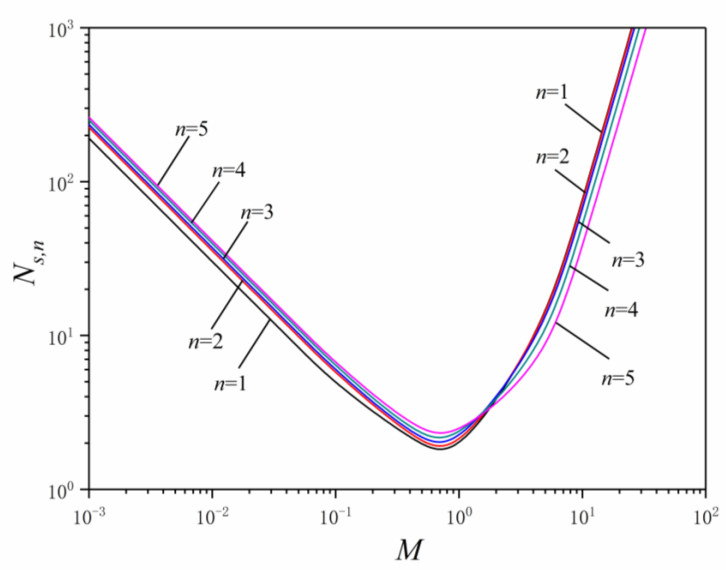
Effects of the dimensionless mass flow rate on EGN.

The relationships between EGN and dimensionless mass flow rate are shown in Figure 7. As the dimensionless mass flow rate increases, the EGN of LVCs with any order first decreases sharply, reach the minimum near M=1 and then increases sharply. When the dimensionless mass flow rate M is less than 2, the EGN of the first order LVCs is the smallest and that of the fifth order is the largest, the EGN of LVCs increases as the order of LVC increases. When the dimensionless mass flow rate M is greater than 2, the EGN of LVCs decreases gradually as the order of LVC increases. 

The characteristics of $N_{s}{}_{,n}$ versus M and $\alpha_{i}$ are listed in Table 3. The EGN of the first to fifth order LVCs with optimal angles $\alpha_{opt,i}$ significantly decrease compared with that with fixed angles $\alpha_{i}$, which is significantly more when the order of LVCs is higher. 

## 5. Conclusions

Based on the entropy generation minimization principle and constructal theory, the constructal optimizations for first to fifth order LVCs with turbulent convection heat transfer by taking the angles at bifurcations as the design variables were conducted with the constraints of fixed vascular areas and the total channel volumes. The analytical expressions of the dimensionless total entropy generation rate and EGN of LVCs with any order versus dimensionless mass flow rate were derived, and the optimal angles of LVCs with first to fifth order were obtained.

(1) The dimensionless total entropy generation rate of LVCs with any order can be significantly decreased by optimizing the angles of LVCs. From the first to fifth order, the dimensionless total entropy generation rate of LVCs with optimal angles were 10.65%, 24.54%, 43.75%, 66.99% and 93.67% smaller than those with fixed angles (α=45.0°), respectively. As the order of LVCs is higher, the dimensionless total entropy generation rate of LVCs decreases significantly more.
(2) Based on the minimum dimensionless total entropy generation rate, as the dimensionless mass flow rate increases, the optimal angles of LVCs with any order remain unchanged first, then the optimal angles of LVCs at the entrance increase, and the other optimal angles of LVCs decrease continuously and finally tend to respective stable values. The optimal angles of LVCs continue to increase from the entrance to the outlet, i.e., the LVCs with a certain order gradually spread out from the root to the crown.
(3) As the dimensionless mass flow rate increases, the dimensionless total entropy generation rate and EGN of LVCs with turbulent convection heat transfer decrease first and then increase sharply. There is optimal dimensionless mass flow rate M can make the dimensionless total entropy generation rate and EGN of LVCs with any order obtain their respective minimums.
(4) The dimensionless total entropy generation rate of LVCs increases gradually as the order of LVC increases for the same dimensionless mass flow rate M. When the dimensionless mass flow rate M is less than 2, the EGN of LVCs increases as the order of LVC increases; however, when the dimensionless mass flow rate M is greater than 2, this is simply reversed.

## Figures and Tables

**Table 1:** Characteristics of $\alpha_{\mathrm{opt},i}$ versus M.

		$\alpha_{\mathrm{opt},0}/{}^{\circ}$	$\alpha_{\mathrm{opt},1}/{}^{\circ}$	$\alpha_{\mathrm{opt},2}/{}^{\circ}$	$\alpha_{\mathrm{opt},3}/{}^{\circ}$	$\alpha_{\mathrm{opt},4}/{}^{\circ}$	$\alpha_{\mathrm{opt},5}/{}^{\circ}$
M=0.1	n=1	78.0	21.3				
	n=2	87.0	78.0	8.6			
	n=3	88.0	86.0	77.8	5.6		
	n=4	88.6	87.8	86.0	77.0	3.6	
	n=5	88.8	88.8	88.8	87.0	77.8	1.8
M=1	n=1	72.6	27.2				
	n=2	81.6	69.1	17.2			
	n=3	86.2	80.6	66.1	11.6		
	n=4	88.2	86.2	79.1	61.8	9.1	
	n=5	88.8	88.8	85.2	77.1	61.6	6.6
M=5	n=1	49.9	42.9				
	n=2	60.0	47.2	40.6			
	n=3	60.8	54.4	48	39.8		
	n=4	66.2	59.0	52.2	46.2	39.4	
	n=5	72.6	65.4	59.0	52.2	46.2	37.6

**Table 2:** Characteristics of ${\tilde{S}}^{*}{}_{gen,n}$ versus M and $\alpha_{i}$.

	M=0.1				M=1				M=5			
	$\alpha_{i}=45{}^{\circ}$	$\alpha_{i}=60{}^{\circ}$	$\alpha_{i}=75{}^{\circ}$	$\alpha_{i}=\alpha_{opt,i}$	$\alpha_{i}=45{}^{\circ}$	$\alpha_{i}=60{}^{\circ}$	$\alpha_{i}=75{}^{\circ}$	$\alpha_{i}=\alpha_{opt,i}$	$\alpha_{i}=45{}^{\circ}$	$\alpha_{i}=60{}^{\circ}$	$\alpha_{i}=75{}^{\circ}$	$\alpha_{i}=\alpha_{opt,i}$
${\tilde{S}}^{*}{}_{gen,1}$	23.918	24.973	29.448	21.105	4.059	4.301	5.539	3.701	25.373	32.158	80.325	25.215
${\tilde{S}}^{*}{}_{gen,2}$	38.356	40.048	47.223	27.710	6.468	6.843	8.747	5.193	36.880	46.706	116.439	36.407
${\tilde{S}}^{*}{}_{gen,3}$	58.785	61.377	72.374	33.715	9.788	10.330	13.003	6.809	45.311	57.264	142.027	44.202
${\tilde{S}}^{*}{}_{gen,4}$	87.645	91.510	107.905	39.582	14.417	15.175	18.810	8.633	51.473	64.840	159.503	49.689
${\tilde{S}}^{*}{}_{gen,5}$	128.261	133.917	157.910	45.052	20.888	21.939	26.845	10.785	56.317	70.612	171.657	53.726

**Table 3:** Characteristics of $N_{s}{}_{,n}$ versus M and $\alpha_{i}$.

	M=0.1				M=1				M=5			
	$\alpha_{i}=45{}^{\circ}$	$\alpha_{i}=60{}^{\circ}$	$\alpha_{i}=75{}^{\circ}$	$\alpha_{i}=\alpha_{opt,i}$	$\alpha_{i}=45{}^{\circ}$	$\alpha_{i}=60{}^{\circ}$	$\alpha_{i}=75{}^{\circ}$	$\alpha_{i}=\alpha_{opt,i}$	$\alpha_{i}=45{}^{\circ}$	$\alpha_{i}=60{}^{\circ}$	$\alpha_{i}=75{}^{\circ}$	$\alpha_{i}=\alpha_{opt,i}$
$N_{s}{}_{,1}$	10.357	10.063	9.017	4.807	1.758	1.733	1.696	1.585	11.528	13.553	25.672	10.987
$N_{s}{}_{,2}$	11.960	11.621	10.412	5.656	2.017	1.986	1.928	1.711	11.499	12.958	24.594	10.937
$N_{s}{}_{,3}$	14.230	13.826	12.388	5.904	2.369	2.327	2.226	1.871	10.968	12.910	24.311	10.089
$N_{s}{}_{,4}$	17.253	16.764	15.020	6.303	2.838	2.780	2.618	2.061	10.133	11.878	22.202	8.217
$N_{s}{}_{,5}$	21.209	20.608	18.464	6.579	3.454	3.376	3.139	2.217	9.313	10.866	20.071	6.528

## Data Availability

Not applicable.

## Nomenclatures

A	areas (m^2^)
$B_{0}$	coefficient
$B_{0}^{\prime }$	coefficient
*D*	diameter (m)
*d*	distance between adjacent outlets (m)
*H*	height (m)
*k*	thermal conductivity (W·(m·K)^−1^)
*L*	length (m)
M	dimensionless mass flow rate
$\dot{m}$	mass flow rate (kg·s^−1^)
$N_{s}$	dimensionless entropy generation number
$q^{\prime }$	linear heat flux (W·m^−1^)
${\dot{S}}_{gen}$	entropy generation rate (J·(K·s)^−1^)
${\tilde{S}}_{gen}$	dimensionless entropy generation rate
*T*	temperature (K)
*V*	volume (m^3^)
**Greek letters**	
α	angel (°)
ρ	density (kg·m^−3^)
ν	kinematic viscosity (m·s^−2^)
**Superscripts**	
*~*	dimensionless
${}^{*}$	transform of physical quantity
**Subscripts**	
*i*	channel rank
in	inlet
n	number of construction orders
out	outlet
opt	optimal
**Abbreviation**	
EGN	entropy generation number
EGR	entropy generation rate
LVC	line-to-line vascular channel
Nu	Nusselt number
Pr	Prandtl number
